# Absent sleep EEG spindle activity in GluA1 (Gria1) knockout mice: relevance to neuropsychiatric disorders

**DOI:** 10.1038/s41398-018-0199-2

**Published:** 2018-08-14

**Authors:** Gauri Ang, Laura E. McKillop, Ross Purple, Cristina Blanco-Duque, Stuart N. Peirson, Russell G. Foster, Paul J. Harrison, Rolf Sprengel, Kay E. Davies, Peter L. Oliver, David M. Bannerman, Vladyslav V. Vyazovskiy

**Affiliations:** 1 Department of Physiology, Anatomy and Genetics, University of Oxford, Parks Road, Oxford, OX1 3PT UK; 2 Department of Experimental Psychology, University of Oxford, South Parks Road, Oxford, OX1 3UD UK; 3 Sleep and Circadian Neuroscience Institute, Oxford Molecular Pathology Institute, Sir William Dunn School of Pathology, South Parks Road, Oxford, OX1 3RE UK; 4 Department of Psychiatry, University of Oxford, Warneford Hospital, Oxford, OX3 7JX UK; 5 Oxford Health NHS Foundation Trust, Warneford Hospital, Oxford, OX3 7JX UK; 6 Research Group of the Max Planck Institute for Medical Research at the Inst. for Anatomy and Cell Biology of the University Heidelberg, INF 307, D-69120 Heidelberg, Germany

## Abstract

Sleep EEG spindles have been implicated in attention, sensory processing, synaptic plasticity and memory consolidation. In humans, deficits in sleep spindles have been reported in a wide range of neurological and psychiatric disorders, including schizophrenia. Genome-wide association studies have suggested a link between schizophrenia and genes associated with synaptic plasticity, including the *Gria1* gene which codes for the GluA1 subunit of α-amino-3-hydroxy-5-methyl-4-isoxazolepropionic acid (AMPA) receptor. *Gria1*^*−/−*^ mice exhibit a phenotype relevant for neuropsychiatric disorders, including reduced synaptic plasticity and, at the behavioural level, attentional deficits leading to aberrant salience. In this study we report a striking reduction of EEG power density including the spindle-frequency range (10–15 Hz) during sleep in *Gria1*^*−/−*^ mice. The reduction of spindle-activity in *Gria1*^*−/−*^ mice was accompanied by longer REM sleep episodes, increased EEG slow-wave activity in the occipital derivation during baseline sleep, and a reduced rate of decline of EEG slow wave activity (0.5–4 Hz) during NREM sleep after sleep deprivation. These data provide a novel link between glutamatergic dysfunction and sleep abnormalities in a schizophrenia-relevant mouse model.

## Introduction

EEG spindles are oscillatory events^1^ that occur predominantly during non-rapid eye movement (NREM) sleep^2^, and have been described in several mammalian species^3^. Spindles are generated within the reticular thalamic nuclei, where neurons typically exhibit a bursting discharge pattern at 7–14 Hz frequencies^4–7^. The rhythmic hyperpolarisation of thalamocortical neurons leads to rebound spike bursts, which are transferred to the neocortex as spindles^8^. Spindle-activity has been extensively studied with respect to numerous processes including brain development^9^, ageing^10^, cortical and behavioural arousal^11,12^, processing of external stimuli^13–16^, attention^17–19^, cognitive performance^20^ and the consolidation of freshly encoded information^21–25^, including hippocampal-to-neocortical information transfer during sleep^26–29^.

The notion of a mechanistic relationship between sleep spindle activity and brain function is supported by the consistent observation that sleep spindles are reduced or have altered dynamics in neuropsychiatric disorders^2,30,31^. Individuals with schizophrenia have been shown to have reduced spindle power density, duration, and number, while evidence suggests that these changes are unlikely due to antipsychotic medication^30,32–35^. Notably, reduced spindle activity is also found in heathy first-degree relatives of patients with schizophrenia, suggesting a genetic contribution to this effect^36^. Yet the mechanisms underlying spindle dysfunction in schizophrenia are unclear.

One possibility is that reduced spindle activity could reflect abnormal glutamatergic neurotransmission and impaired synaptic plasticity, which are widely believed to be core features of the disorder^37,38^. Theories have tended to focus on N-methyl-D-aspartate (NMDA) receptor hypofunction as being the main cause of glutamatergic dysfunction in schizophrenia^39–42^. But recent genome-wide association studies (GWASs) have also established the *GRIA1* locus, which codes for the human AMPA receptor GluA1 subunit, to have a genome-wide significant association with schizophrenia^43^. In combination with earlier work which showed subjects with schizophrenia had reduced GluA1 expression in the hippocampus and thalamus^44,45^, these findings point to an important role for GluA1 in the aetiology of the disorder. Notably, GluA1 deficient mice (*Gria1*^*−/−*^ mice) exhibit impaired hippocampal synaptic plasticity^46,47^, and extensive behavioural studies have revealed deficits in habituation and attention, relevant for psychosis aetiology^48–51^. Thus, mice lacking GluA1 represent an important model for studying glutamatergic dysfunction in psychotic disorders, including schizophrenia. Moreover, GluA1 dysfunction may also contribute to a number of other neuropsychiatric and neurological conditions, including Rett syndrome^52^, fragile X syndrome^53^, temporal lobe epilepsy^54^ and limbic encephalitis^55^.

We therefore investigated the role of the GluA1 subunit in the dynamics of cortical EEG activity in spontaneously sleeping mice. We show that *Gria1*^*−/−*^ mice exhibit sleep EEG abnormalities and reduced spindle activity that closely correspond to the phenotype observed in schizophrenic patients. These data provide an important novel link between glutamatergic dysfunction, impaired synaptic plasticity and sleep EEG abnormalities in a schizophrenia-relevant mouse model.

## Materials and methods

### Animals and recording conditions

*Gria1*^*−/−*^ mice were bred by crossing heterozygous *Gria1*^*+/-*^ mice, in the Biomedical Sciences Building, University of Oxford. Genetic construction, breeding, and genotyping were performed as previously described^46^. *N* = 6 male *Gria1*^*−/−*^ mice and *n* = 8 male littermate wild-type mice (WT) aged 7–8 months underwent electroencephalogram (EEG) and EMG recording. The sample size used in this study was based on previous similar studies which investigated sleep and sleep EEG in mice carrying a null mutation of the gene of interest^56,57^. For the duration of the experiment mice were individually housed in custom-made clear plexiglass cages (20.3 × 32 × 35 cm) with free access to running wheels (Campden Instruments, Loughborough, UK, 20 × 23 cm, wheel diameter 14 cm, bars spaced 1 cm apart) on a 12:12 h light-dark (12:12 LD) cycle. Cages were placed in ventilated, sound-attenuated Faraday chambers (Campden Instruments, Loughborough, UK, up to two cages per chamber). The animals were placed in recording chambers in a pseudo-random order according to their genotype; the investigator was not blinded to group allocation. Each chamber had an LED lamp illuminating the chamber at approximately 200 lux during the light phase of the 12:12 LD cycle. Room temperature and relative humidity were maintained at 22 ± 1 °C and 60 ± 10%, respectively. Food and water were available ad libitum throughout all studies. All procedures were performed in accordance with the United Kingdom Animals (Scientific Procedures) Act of 1986 and the University of Oxford Policy on the Use of Animals in Scientific Research (PPL 70/7483). All experiments were approved by the University of Oxford Animal Welfare and Ethical Review Board. *Gria1*^*−/−*^ mice are available from Jackson labs, stock #019011 (https://www.jax.org/strain/019011).

### Surgical procedure and experimental design

**Fig. 1:**
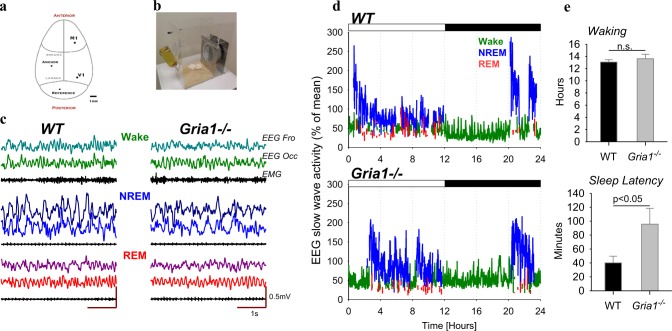
**a** Schematic depiction of the positions of the EEG electrodes placed above the primary motor cortex (M1, frontal derivation) and above the primary visual cortex (V1, occipital derivation), and the position of reference and anchor screws. **b** Animals were individually housed in custom-made home cages, shown on the photograph, providing continuous free access to a running wheel, inside ventilated, sound-attenuated chambers provided with autonomous light-dark (12:12) control. **c** Representative EEG traces recorded from the frontal and the occipital derivations and the EMG during waking, NREM and REM sleep in one individual WT mouse and one *Gria1*^*−/−*^ mouse. **d** Hypnograms of a representative wild-type (WT) and *Gria1*^*−/−*^ mouse. 24-h profile of EEG slow-wave activity (SWA, EEG power between 0.5–4.0 Hz, represented as % of 24-h mean) recorded in the frontal cortex, is colour-coded according to the vigilance state (wake = green, NREM sleep = blue, REM sleep = red). The bar on the top of the panels depicts 12-h light and 12-h dark periods. **e** Top: average amount of wakefulness during 24-h baseline period. Mean values, SEM, *n* = 8 (WT) and *n* = 6 (*Gria1*^*-/-*^). Bottom: Average latency from the light onset until the occurrence of first consolidated sleep period >3 min. Mean values, SEM. The *p*-value above indicates significant difference between the genotypes (*p* < 0.05, Wilcoxon rank sum test)

The animals underwent cranial surgery to implant custom-made EEG and EMG headmounts. Each headmount was composed of three stainless steel screw electrodes and two stainless steel wires (shaft diameter 0.86 mm, InterFocus Ltd, Cambridge, UK), attached to an 8-pin surface mount connector (8415-SM, Pinnacle Technology Inc, Kansas, USA). Surgical procedures were carried out under isoflurane anaesthesia (5% for induction, 1.5–2.5% for maintenance) using aseptic surgical techniques. Throughout the surgical procedure, animals were head-fixed using a stereotaxic frame (David Kopf Instruments, California, USA); Viscotears liquid gel (Alcon Laboratories Limited, Hemel Hempstead, UK) was applied at regular intervals to protect the eyes. An incision was made along the midline of the head and the skull was cleaned with 3% hydrogen peroxide followed by saline. A high-speed drill (carbon burr drill bits, 0.7 mm, InterFocus Ltd, Cambridge, UK) was used to drill holes in the skull. Two of the headmount screws were implanted epidurally over the frontal (motor area, M1, anteroposterior (AP) + 2 mm, mediolateral (ML) 2 mm) and occipital (visual area, V1, A*P* −3.5 to 4 mm, ML + 2.5 mm) cortical regions (Fig. 1a). An additional screw was implanted over the cerebellum to act as a reference, and an anchor screw was attached to the skull contralaterally to the frontal screw to stabilise the head implant. Two stainless steel wires were inserted either side of the nuchal muscle to record EMG. All screws and the headmount wires were secured using dental cement (Associated Dental Products Ltd, Swindon, UK). Overall, this recording configuration provided two EEG derivations (frontal (F) vs. cerebellum and occipital (O) vs. cerebellum) and one EMG derivation. All animals were administered saline and were maintained on thermal support throughout surgery and for the immediate hours following. Analgesics were administered preoperatively (Metacam 1–2 mg/kg, s.c., meloxicam, Boehringer Ingelheim Ltd, Bracknell, UK) and for at least three post-operative days (orally together with mashed chow). A minimum 2-week recovery period was permitted prior to cabling the animals for recording. Mice were habituated to both the running wheel and recording cable for a minimum of four days before the recording began. Diurnal entrainment was verified by analysis of wheel-running activity over this time period. Since the primary aim of this study was to provide the first characterisation of spontaneous sleep and sleep EEG in *Gria1*^*−/−*^ mice, rather than the effects of specific manipulations beyond conventional sleep deprivation, all experiments were carried out under standard laboratory conditions, where mice were kept under a 12:12 LD cycle in the home-cage environment, and not exposed to any behavioural tasks.

### Sleep deprivation

After an undisturbed spontaneous baseline 24-h recording period (beginning at lights-on), the animals were sleep-deprived for 6-h (sleep deprivation, SD) from light onset while polysomnographic recordings were made under constant visual observation. SD was performed in the animal’s home cage, where they had free access to running wheels throughout the procedure, which they used intermittently. Throughout the SD procedure, the animals were regularly provided with various objects, which elicited exploratory behaviour, to mimic naturalistic conditions of wakefulness in an ethologically relevant manner^58–60^. The objects included nesting and bedding material from other cages, wooden blocks, small rubber balls, plastic, metallic, wooden, or paper boxes and tubes of different shape and colour. To avoid stress, the animals were habituated to the exposure of these novel objects prior to the experiment. Subsequently, the animals were left undisturbed for the rest of the 24-h period, and analysed as the recovery period. During SD, the animals were successfully kept awake (WT: 98.6 ± 0.6%, *Gria1*^*−/−*^: 99.8 ± 0.1% of 6 h).

### Statistics

**Fig. 2:**
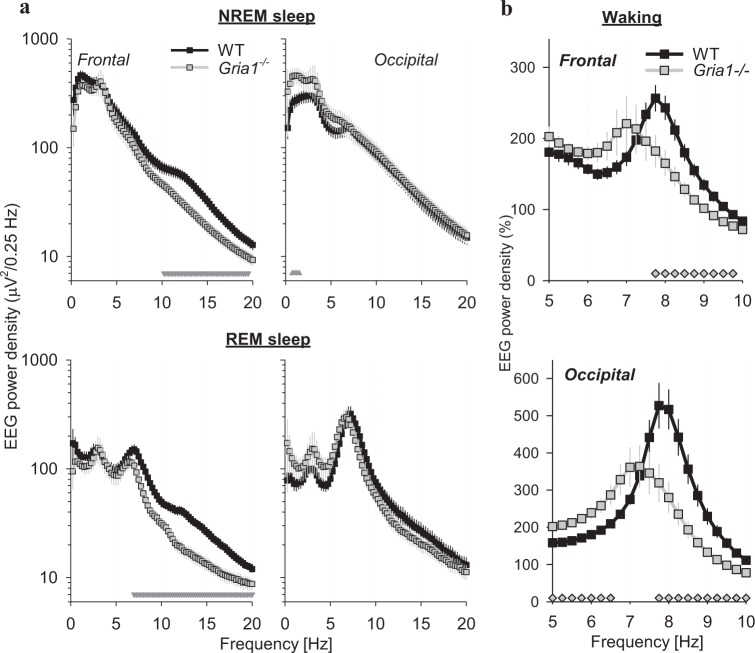
**a** EEG power density during NREM sleep (top) and REM sleep (bottom) in the frontal (left) and occipital (right) derivation. Mean values, SEM, *n* = 8 (WT) and *n* = 6 (*Gria1*^*-/-*^); NREM: genotype*derivation*bin interaction: F(80,960) = 4.582, *p* < 0.001; REM: genotype*derivation*bin interaction: F(80,960) = 3.612, *p* < 0.001; three-way repeated-measures ANOVA on log-transformed values; note that the data for the frontal and occipital derivation are shown in separate plots for clarity). The triangles below depict frequency bins where EEG spectra differed significantly between the genotypes (*p* < 0.05, post hoc Bonferroni test). **b** EEG power density during waking in the frontal (left) and occipital (right) derivation. Mean values, SEM (WT: *n* = 8, *Gria1*^*−/−*^: *n* = 6), are shown for the frequency range encompassing theta-activity, and the values are expressed as % of mean EEG power density across all frequencies from 0.5 to 20 Hz excluding the theta-frequency range (genotype*derivation*bin interaction: F(80,960) = 2.455, *p* < 0.001, three-way repeated-measures ANOVA on log-transformed values). The symbols below depict frequency bins where EEG spectra differed significantly between the genotypes (*p* < 0.05, Bonferroni post hoc test)

**Fig. 3:**
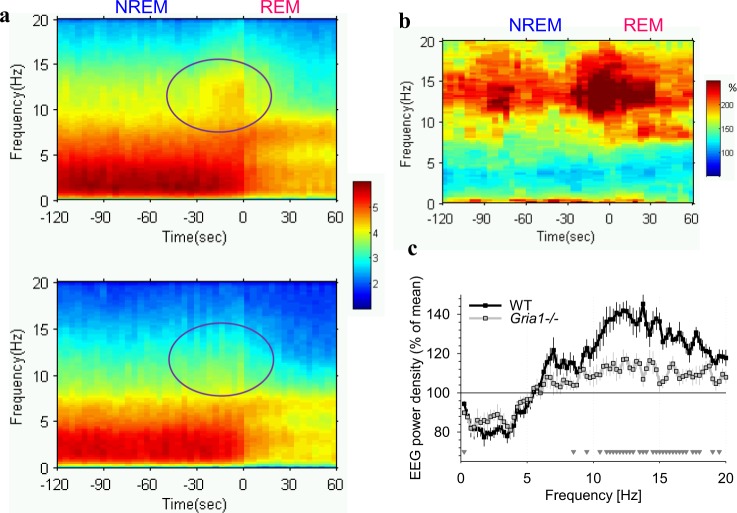
**a** Dynamics of EEG spectra in the frontal derivation are shown for the transition from NREM sleep to REM sleep for the 24-h baseline. Only REM episodes > 1 min were included in this analysis (33.6 ± 1.2 and 28.8 ± 2.4 episodes per animal, WT and *Gria1*^*−/−*^ respectively). 2 min prior to the transition ( = 0) and 1 min after the transition is illustrated based on 4-s epochs (*x*-axis: in seconds). Mean values (top panel: WT, *n* = 8, bottom panel: *Gria1*^*−/−*^, *n* = 6). EEG power density is colour-coded according to the log scale (µV^2^/0.25 Hz) on the right. Note a surge of EEG power in spindle-frequency range immediately prior to transition in WT mice, which is attenuated in *Gria1*^*−/−*^ mice (circled). **b** The difference between WT and *Gria1*^*−/−*^ is represented as %. For this analysis the average spectral values calculated for each 4-s epoch in WT mice are expressed as percentage from the corresponding spectral values in *Gria1*^*−/−*^ mice. **c** Mean frontal EEG power density during the last 32 s of NREM sleep prior to transition to REM sleep. Mean values, SEM (WT: *n* = 8, *Gria1*^*−/−*^: *n* = 6) for each frequency bin expressed as % of mean EEG power over all artefact-free 4-s epochs in NREM sleep during 24 h in the corresponding bin (genotype*bin interaction: F(80,960) = 4.710, *p* < 0.001, two-way repeated-measures ANOVA). Triangles below the curves depict frequency bins, where EEG power during the last 32 s before NREM-REM transition differed significantly between genotypes (*p* < 0.05, post hoc Bonferroni test)

**Fig. 4:**
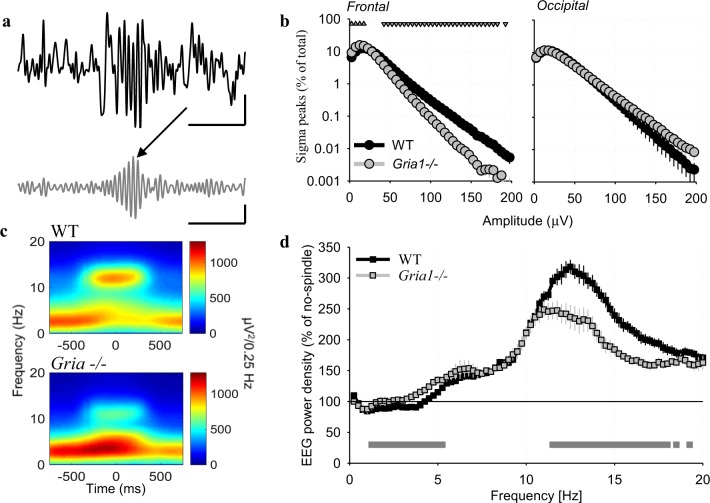
**a** Individual 4-s record of the frontal EEG with a spindle-event. Top trace: raw EEG signal; bottom trace: EEG signal filtered between 10 and 15 Hz. Scale bars: horizontal, 1 s, vertical: 100 µV. **b** Distribution of EEG-waves filtered in sigma-frequency band (10–15 Hz) as a function of their amplitude. The number of waves is plotted against progressively increasing amplitude and expressed as % of the total number of waves. Mean values, WT: *n* = 8, *Gria1*^*−/−*^: *n* = 6, SEM (genotype*derivation*bin interaction: F(39,468) = 3.813, *p* < 0.001, three-way repeated-measures ANOVA). Triangles above the curves denote amplitude bins, where the distributions differed significantly between the genotypes (*p* < 0.05, post hoc Bonferroni test). Scale bars: horizontal, 1 s, vertical: 100µV. **c** Distribution of spectral power <20 Hz around the midpoint of individual spindle events: top: WT mice, bottom: *Gria1*^*−/−*^ mice. **d** Mean EEG power density in the frontal derivation during epochs with detected spindle events. Mean values, SEM (WT: *n* = 8, *Gria1*^*−/−*^: *n* = 6) for each frequency bin expressed as % of mean EEG power during NREM sleep epochs without detected spindles (genotype*bin interaction: F(80, 960) = 17.681, *p* < 0.001, two-way repeated-measures ANOVA). Grey line below the curves depicts frequency bins, where relative EEG power density differed between the genotypes (*p* < 0.05, post hoc Bonferroni test)

**Fig. 5:**
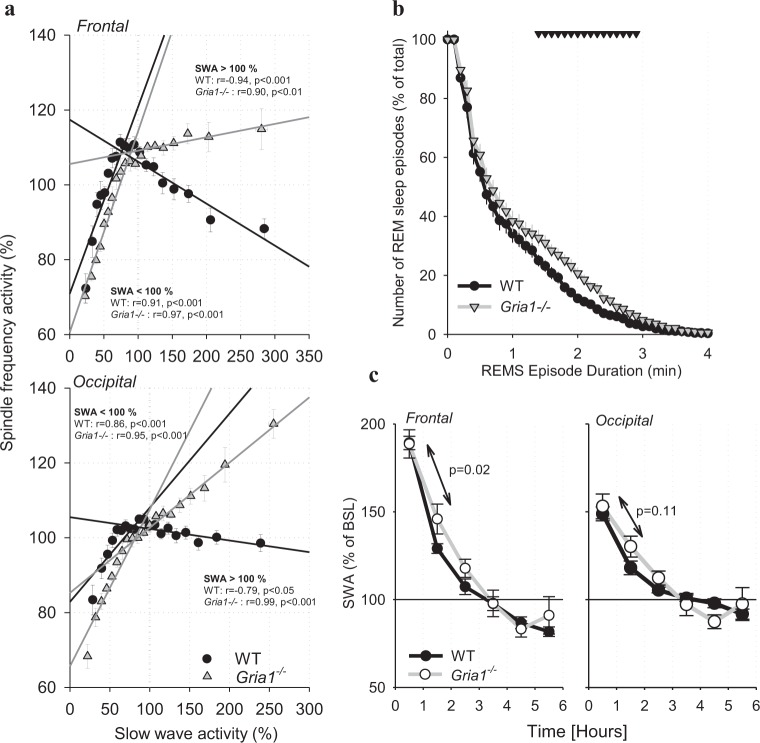
**a** The relationship between EEG slow-wave activity (SWA, 0.5–4.0 Hz) and EEG spindle-frequency activity (SFA, 10–15 Hz) during baseline. All 4-s epochs in artefact-free NREM sleep occurring during the 24-h baseline were subdivided into twenty 5% bins as a function of SWA sorted from lowest to highest values, and corresponding SFA values were averaged for the same epochs prior to calculating means between animals (shown as symbols, *n* = 8 for WT and *n* = 6 for *Gria1*^*−/−*^, SEM). Straight lines depict linear regressions separately for epochs with SWA < 100% ( = below the mean value of SWA over the 24-h period) and <100% ( = above the mean value of SWA over the 24-h period). *R* and *p*-values correspond to Pearson’s product moment correlation. Note that when SWA is low (<24-h mean), it correlates positively with SFA in both genotypes, but the correlation is negative for SWA values >100% for WT mice only. **b** Survival analysis of REM sleep continuity (genotype*bin interaction: F(50, 600) = 2.016, *p* < 0.001, two-way repeated-measures ANOVA). The *x*-axis represents the episode durations in minutes and the *y*-axis represents the percentage of episodes remaining in REM state for that episode length or longer. Mean values, SEM. The triangles above the curves denote significant differences between the genotypes (*p* < 0.05, post hoc Bonferroni test). **c** Time course of NREM EEG slow-wave activity (SWA, 0.5–4 Hz) in the frontal derivation after 6-h sleep deprivation (SD). SWA values are expressed as % of mean 24-h baseline value (genotype*bin interaction: F(4,48) = 5.045, *p* = 0.002, two-way repeated-measures ANOVA). Mean values, SEM. The values inside the panels are *p*-values for the differences in the decline rate from the 1st to the 2nd 1-h interval between the genotypes (Wilcoxon rank sum test)

Statistical analyses were performed with SPSS 24.0 (IBM, Armonk, New York). Repeated-measures ANOVAs were run on data with genotype (WT or *Gria1*^*−/−*^ mice) as a between-subject factor, and derivation (frontal, occipital) or frequency bins (Figs. 2–3), sigma-peaks amplitude bins (Fig. 4) or REM episode duration bins (Fig. 5) as within-subjects factors. Where significant interactions between factors were found, post hoc Bonferroni tests were performed. Since EEG spectral power values are not normally distributed, the statistical comparisons were performed on log-transformed data^56^.

### Signal processing

Data acquisition was performed using the Multi-channel Neurophysiology Recording System (TDT, Alachua FL, USA)^61^. EEG and EMG data were collected at a sampling rate of 256.9 Hz (filtered between 0.1 and 100 Hz), amplified (PZ5 NeuroDigitizer pre-amplifier, TDT Alachua FL, USA) and stored on a local computer. Data were resampled offline at a sampling rate of 256 Hz. Signal conversion was performed using custom-written MatLab (The MathWorks Inc, Natick, MA, USA) scripts and was then transformed into European Data Format (EDF) using open source Neurotraces software. For each 24-h recording, EEG power spectra were computed by a Fast Fourier Transform (FFT) routine for 4-s epochs, with a 0.25 Hz resolution (SleepSign Kissei Comtec Co, Nagano, Japan).

### Scoring of vigilance states

Vigilance states were scored offline through visual inspection for consecutive 4-s epochs (SleepSign, Kissei Comtec Co, Nagano, Japan). Two EEGs (frontal and occipital), EMG and running wheel activity were displayed simultaneously to aid vigilance state scoring. Vigilance states were classified as wake (low voltage, high-frequency irregular EEG pattern, dominated by theta-activity (6–9 Hz), especially in the occipital EEG during running, with a high level or phasic EMG activity), NREM sleep (presence of slow waves, a signal of a high amplitude and low frequency), or REM sleep (low voltage, higher frequency EEG dominated by theta-activity in the occipital derivation, with a low level of EMG activity). Epochs during which the EEG signals were contaminated by artefacts caused by eating, drinking or gross movements, were removed from spectral analysis (WT: 3.6 ± 0.8% of total recording time, 98.6 ± 0.9 during wake; *Gria1*^*−/−*^: 1.9 ± 0.3% of total recording time, 96.8 ± 2.5 during wake). The onset of individual NREM sleep episodes was defined by the first occurrence of slow waves (0.5–4 Hz) in at least one of the two EEG channels, accompanied by the absence of phasic EMG activity recorded from the nuchal (back neck) muscle. For NREM-REM transitions analyses, we included REM sleep episodes, which were at least 1 min long, and were preceded by NREM sleep episodes lasting at least 2 min. To calculate sleep latency after light onset, we included consolidated sleep periods, comprising both NREM and REM sleep, lasting at least 3 min.

### Spindle detection

Spindle events were clearly discernible in all WT mice through inspection of the raw EEG. These occurred during sleep, especially during NREM sleep. Spindles were characterised as bursts of EEG activity at frequencies between ~10 and 15 Hz, and were mostly encountered in the frontal EEG derivation. An algorithm for automatic detection of spindles was developed based on an amplitude threshold obtained for each individual mouse (WT: 48.3 ± 2.7, *Gria1*^*−/−*^: 37.8 ± 2.5 µV, *p* < 0.05, Wilcoxon signed-rank test), and was applied to the band-pass filtered EEG. To calculate the threshold, we first filtered the EEG signal between 10 and 15 Hz, and detected all positive sigma-frequency waves in the resulting signal. Next, we heuristically determined a threshold for spindle detection for each individual animal and separately for each genotype. This was defined as the mean + one standard deviation across maximal amplitudes of all positive sigma-frequency waves in NREM sleep. Spindle events lasting at least 500 ms were included in the final analyses. To investigate the relationship between spindles and other EEG frequencies, spectrograms were computed for each detected spindle using 2 s intervals centred on the spindle midpoint. To this end, a short-time Fourier transform was applied using a 500 ms window length with a 95% overlap at a frequency resolution of 0.25 Hz (Fig. 4c).

## Results

### EEG spectral power in the spindle-frequency range is decreased in *Gria1*^*−/−*^ mice

Both genotypes showed a typical light:dark distribution of vigilance states, with wakefulness predominantly occurring during the dark period and sleep mostly occurring during the light phase (individual representative examples: Fig. 1). While the total daily amount of waking did not differ between genotypes (Fig. 1d, top, Table 1), the *Gria1*^*−/−*^ animals showed a significantly longer latency from light onset until the first consolidated period of sleep (Fig. 1d, bottom), which was also reflected in a higher proportion of waking during the light period (Table 1).

**Table 1:** Vigilance states, NREM and REM sleep episodes in Gria1^*−/−*^ and wild-type mice (Gria1^ + / + ^)

	*Gria1* ^*+* */* *+* ^					*Gria1* ^-/-^				
BASELINE	WAKING	NREMS	REMS	NREM episodes	REM episodes	WAKING	NREMS	REMS	NREM episodes	REM episodes
Light	31.9 (2.0)	54.5 (2.3)	10.4 (0.6)	79.3 (4.7)	84.1 (7.2)	41.8 (4.4) ^a^	47.8 (3.7)	8.1 (0.6)°	60.3 (4.2) ^a^	56.0 (6.4) ^a^
Dark	77.1 (2.9)	19.7 (2.5)	2.2 (0.4)	22.5 (4.4)	16.4 (2.7)	72.0 (4.0)	23.5 (3.3)	3.2 (0.6)	26.5 (4.2)	19.8 (4.0)
24-h	54.5 (1.6)	37.2 (1.9)	6.3 (0.3)	101.5 (7.3)	100.5 (8.2)	56.8 (3.1)	35.7 (2.5)	5.6 (0.4)	86.8 (6.9) °	75.8 (7.8) °
SLEEP DEPRIVATION	98.8 (0.6)	1.2 (0.5)	0.0	1.0 (0.6)	0.0	99.8 (0.1)^a^	0.1 (0.1)^a^	0.0	0.0	0.0
RECOVERY Light	27.8 (4.5)	58.4 (4.0)	10.9 (0.6)	33.8 (1.6)	39.3 (2.0)	25.3 (2.5)	61.5 (2.1)	10.6 (0.4)	32.0 (2.0)	31.7 (2.3) °
Dark	73.8 (2.3)	21.7 (1.9)	3.3 (0.4)	24.1 (4.1)	21.4 (4.2)	62.0 (4.7) ^a^	31.1 (3.9) °	5.2 (0.7)^a^	41.2 (4.7) ^a^	34.8 (4.6)

Mean values (SEM in parenthesis, *Gria1*^+/+^: n=8, *Gria1*^-/-^: n=6) of waking, NREM sleep (NREMS) and REM sleep (REMS) expressed as percentage of recording time, and the total number of NREMS and REMS episodes, shown for the 12-h light and dark period of baseline day, 6-h sleep deprivation (SD) and 18-h recovery. SD began at light onset. Differences between genotypes: ° p<0.1, ^a^ p<0.05, Wilcoxon rank sum test

EEG spectral analysis during NREM sleep revealed that *Gria1*^−/−^ mice had a substantially lower frontal EEG power density at frequencies above 10 Hz (Fig. 2a, top), as compared to WT control animals. In contrast, *Gria1*^−/−^ mice had higher EEG power density in lower frequencies (0.75–1.5 Hz) in the occipital derivation as compared to WT mice (Fig. 2a). The regional difference in SWA during NREM sleep was substantially reduced in *Gria1*^*−/−*^ mice: while SWA was approximately 50% higher in the frontal derivation as compared to the occipital cortex in WT animals, individual *Gria1*^*−/−*^ mice were highly variable in this respect, resulting in, on average, similar values of SWA between the frontal and the occipital derivation (WT: frontal 395.1 ± 47.5 µV^2^/0.25 Hz, occipital 271.1 ± 35.2; *Gria1*^*−/−*^: frontal 345.2 ± 41.5, occipital: 406.6 ± 97.4 µV^2^/0.25 Hz; frontal as % of occipital: WT 155 ± 17.0%, *Gria1*^*−/−*^: 99.5 ± 15.9%, *p* < 0.05, Wilcoxon rank sum test). EEG power density in *Gria1*^*−/−*^ animals was significantly lower in frequencies > 7 Hz during REM sleep (Fig. 2a, bottom). During baseline recordings in the home-cage environment, wakefulness EEG power density did not differ significantly between the genotypes, with the exception of one frequency bin (15.75 Hz), where spectral power was significantly higher in WT animals (not shown). However, it was apparent that the theta-peak was substantially more pronounced in the wake EEG spectra of WT mice, as compared to *Gria1*^*−/−*^ mice (Fig. 2b).

Since EEG spindles in rodents are especially prominent at the transition from NREM to REM sleep^56,62–64^, we calculated an average spectrogram over 3-min intervals, which included the last 2 min of NREM sleep and the first 1 min of the following episode of REM sleep. As expected, EEG power density between 10 and 15 Hz showed a surge during the last 30 s before REM sleep, but this increase was only discernible in WT animals (Fig. 3a). Calculating the difference between the spectrograms in WT and *Gria1*^*−/−*^ mice revealed ~2–3 fold higher values of EEG power before the transition in WT animals as compared to *GluA1*^*−/−*^ mice (Fig. 3b). This result was confirmed when we calculated EEG power spectra specifically during the last 32 s before REM sleep onset, which resulted in WT animals having significantly higher EEG power values in higher frequencies encompassing the spindle-frequency range (10–15 Hz, Fig. 3c). In contrast, EEG spectral power prior to NREM-REM transitions was not significantly different between genotypes in lower frequencies. Thus, in summary, the *Gria1*^*−/−*^ genotype is associated with a prominent decrease in EEG power density in frequencies encompassing the spindle-frequency range, which was especially apparent prior to NREM-REM sleep transitions.

### EEG spindle events are absent in *Gria1*^*−/−*^ mice

Since spectral analysis does not allow the separation of phasic spindle oscillations from continuous background EEG activity, the possibility remains that the decrease in EEG power density in *Gria1*^*−/−*^ animals merely represents an overall lower EEG amplitude in the higher frequencies including sigma-frequency band (10-–15 Hz). Therefore, we next investigated the occurrence of individual spindle events. Visual inspection revealed the occurrence of well-defined bursts of activity between 10 and 15 Hz in the frontal derivation of all individual WT animals (Fig. 4a). To quantitatively analyse their occurrence and specific characteristics, we filtered the EEG signal between 10 and 15 Hz (sigma-frequency band, SFB), and detected all positive SFB waves. Plotting the distribution of the amplitudes of all individual SBF waves revealed a noticeable difference in the occurrence of waves above approximately 50 µV, which were significantly less frequent in GluA1 deficient mice (Fig. 4b). We next determined a threshold for spindle detection individually for each animal, defined as the mean plus one standard deviation across all positive sigma-frequency waves during NREM sleep, to detect individual spindle events (see Methods). As expected, the algorithm detected putative spindle events in both genotypes. However, visual inspection of the EEG traces in all individual animals did not reveal distinct well-defined spindle events in *Gria1*^*−/−*^ mice, while these were clearly discernible in WT animals (Fig. 4a). Furthermore, spectrograms centred on the midpoint of individual spindle events revealed that mice lacking GluA1 showed a lack of suppression of EEG power in the slow-wave frequency range in association with spindles (Fig. 4c). This suggests that rather than being true spindle events, the detected ‘spindles’ in *Gria1*^*−/−*^ mice merely reflect non-specific, random variations in background spindle-frequency activity (SFA).

Next, we calculated EEG spectra separately for those 4-s epochs where spindles were detected and those epochs without automatically detected spindles (Fig. 4d). As expected, this analysis revealed substantially enhanced EEG power density in higher frequencies encompassing the spindle-frequency range, as described previously^62^, in both genotypes. However, relative spectral power in frequencies between 11.5 and 18.0 Hz was significantly higher in WT mice as compared to *Gria1*^*−/−*^ animals, suggesting that despite the latter having a lower threshold for spindle detection, the separation between ‘spindle’ and ‘no-spindle’ epochs was suboptimal, with a higher incidence of false positive detections in *Gria1*^*−/−*^ mice.

To assess further the relationship between the dynamics of SFA and SWA, we then calculated the correlation between these two variables by grouping all 4-s epochs in NREM sleep into twenty 5% bins as a function of SWA (Fig. 5a). Previously, similar analysis performed on human sleep EEG has revealed a positive association between spindle characteristics and delta power for epochs with low SWA, while the opposite was apparent (i.e., there was a negative correlation) for epochs with high SWA^65^. Consistently, we found a similar relationship between SFA and SWA, but this was apparent only in WT animals (Fig. 5a). In contrast, SFA and SWA correlated positively in *Gria1*^*−/−*^ mice even for epochs with SWA > mean 24-h value, especially in the occipital derivation. This suggests that the occurrence of EEG activity in the spindle-frequency range in *Gria1*^*−/−*^ mice merely reflects overall global changes in the EEG power, rather than any physiological occurrence of spindle events.

### Prolonged REM sleep and altered dynamics of NREM SWA in *Gria1*^*−/−*^ mice

Given the prominence of spindles prior to NREM-REM transitions, we hypothesised that their reduced occurrence in *Gria1*^*−/−*^ mice may lead to changes in NREM and REM sleep continuity. Furthermore, since characteristic sleep-time dependent dynamics of spindle-activity suggest that they may be involved in homoeostatic regulation of sleep, it is possible that reduced spindle occurrence in *Gria1*^*−/−*^ animals may be associated with altered dynamics of sleep homoeostasis.

Firstly, to assess sleep continuity we calculated the duration of NREM sleep episodes, during the 24-h baseline period. The resulting values were consistent with previously published values in C57BL/6J mice^66^, and similar between the genotypes (WT: 4.7 ± 0.4 min, *Gria1*^*−/−*^: 5.3 ± 0.3 min, n.s.). However, the incidence of NREM sleep episodes was reduced in *Gria1*^*−/−*^ mice, especially during the light period (Table 1). On the other hand, REM sleep episodes were slightly longer in *Gria1*^*−/−*^ mice, as compared to WT controls (1.0 ± 0.04 and 1.1 ± 0.04 min, Wilcoxon rank sum test, *p* < 0.1), while their incidence was also lower (Table 1). To address whether these differences reflect the effects of GluA1 deletion on REM sleep maintenance, we performed survival probability analysis, which allows assessment of how long a specific state continues without interruption^67^. Interestingly, this analysis revealed an increased occurrence of REM sleep episodes exceeding approximately 1 min in *Gria1*^*−/−*^ mice as compared to WT controls (Fig. 5b). Notably, increased tendency to remain in REM sleep once an episode was initiated was associated with a reduced number of REM sleep episodes overall, possibly suggesting that REM sleep maintenance partially compensates for reduced REM sleep time (Table 1).

To address the possibility that REM episode duration is related to spindle activity immediately prior to the transition, we calculated the time course of spindle incidence during the last 1 min before REM sleep onset, separately for REM sleep episodes shorter and longer than 1 min. While spindles were overall more frequent in WT mice as compared to *Gria1*^*−/−*^ animals, especially closer to REM sleep onset, there was no difference between spindle incidence before short and long REM sleep episodes (not shown). Furthermore, correlation analysis did not reveal a significant relationship between the average spindle incidence and the average duration of REM sleep episodes in individual animals of either genotype (Pearson’s correlation, all *p*-values > 0.5). Therefore, it is unlikely that the reduced incidence of spindles in *Gria1*^*−/−*^ mice is associated with a prolongation of REM sleep episodes.

Since spindle activity shows pronounced dynamics within the sleep period, it has been suggested that sleep spindles may be involved in the homoeostatic regulation of sleep^62,68^. Therefore, we hypothesised that the spindle deficit observed in *Gria1*^*−/−*^ mice may be associated with changes in the dynamics of EEG slow-wave activity (SWA, 0.5–4 Hz), which is determined by preceding sleep-wake history^69^. To address this possibility we investigated diurnal changes in EEG SWA during NREM sleep in *Gria1*^*−/−*^ mice and WT controls. We found a typical declining time course of SWA during the baseline 12-h light period (not shown), and a robust increase of SWA after sleep deprivation (SD) in both genotypes (Fig. 5c). The overall change in EEG spectra after SD was similar between WT and *Gria1*^*−/−*^ mice (not shown). However, while the initial value of SWA during the first 1-h interval after SD was virtually identical between the genotypes, we noticed that the value of SWA during the 2nd 1-h interval (i.e. between 1 and 2 h) was consistently higher in *Gria1*^*−/−*^ mice as compared to WT mice in both the frontal and the occipital derivation (mixed-model ANOVA, interaction genotype*time interval, *p* < 0.05 for both derivations). We therefore calculated the change of SWA from the 1st to the 2nd 1-h interval after SD, which revealed a substantially faster decline in SWA in WT mice, as compared to *Gria1*^*−/−*^ animals in the frontal derivation (decrease from the 1st interval to the 2nd interval, in %: WT: 60.1 ± 3.4, *Gria1*^*−/−*^: 42.8 ± 5.7, Wilcoxon rank sum test, *p* < 0.05). This finding suggests that the lack of spindles in GluA1 deficient mice may result in a less restorative sleep, manifested as a reduced efficiency of the initial sleep after sleep deprivation for dissipating sleep pressure.

## Discussion

Here we report a striking reduction of sleep EEG spindle-frequency power concomitant with the absence of clear-cut spindle events in *Gria1*^*−/−*^ mice, a model of glutamatergic dysfunction and impaired synaptic plasticity relevant for schizophrenia. Notably, the effect was specific to the frontal EEG, where spindles in rodents are typically observed^62^, and mostly encompassed frequencies corresponding to spindles (10–15 Hz), while lower frequencies were only marginally affected. In addition, we report longer latencies to sleep after light onset, a prolongation of sustained REM sleep episodes, elevated SWA in the occipital derivation, an abnormal relationship between SFA and SWA across 24-h in the frontal cortex, and a slower rate of decay of SWA after sleep deprivation in *Gria1*^*−/−*^ mice.

These findings are of particular interest given the link between GluA1 and a variety of brain disorders^52–55^, including schizophrenia^30,43,70^. Specifically, several studies have reported reduced spindle activity in human patients with schizophrenia, including in non-medicated subjects^32,33,35,71,72^ and, notably, also in healthy first-degree relatives of patients with schizophrenia^36^. Patients with schizophrenia have been shown to exhibit a whole night deficit in spindle-frequency power (12–16 Hz), which resulted from lower amplitude, duration and incidence of sleep spindles. This has been observed in several cortical regions, including the prefrontal, centroparietal, and temporal areas^35^. Thus, our findings of reduced spindle-frequency power, as well as spindle amplitude and incidence, in *Gria1*^*−/−*^ mice are strikingly reminiscent of the patient phenotype. These data suggest that glutamatergic dysfunction may play a pivotal role in sleep and sleep spindle disruption in schizophrenia.

### Neural mechanisms underlying impaired sleep spindles

Our data provide a novel and important link between glutamatergic dysfunction and abnormal sleep EEG. There are several potential mechanisms, which may underpin this link, including cellular and network mechanisms of spindle generation and synchronisation, or processes pertaining to sleep homoeostasis. Previously, it has been suggested that the deficit in sleep spindles may originate from an impairment in thalamocortical circuitry, with a specific emphasis on the thalamic reticular nucleus (TRN)^71,73,74^. However, while AMPA receptors undoubtedly play an important role in the function of thalamocortical circuitry^75,76^, it remains to be determined whether TRN function is affected in *Gria1*^*−/−*^ mice.

Alternatively, there is evidence that spindle characteristics are regulated not only by activity in the reticular nucleus, but also by corticothalamic feedback control^77–79^, and intracortical and thalamocortical connectivity^79–81^. It has been shown that the TRN is not enriched in GluA1 subunits, at least in rats^82^. Therefore, it is possible that local inhibitory circuitry in the TRN remains intact in *Gria1*^*−/−*^ mice, and rather the reduced spindle activity reflects a wider network phenomenon. For example, it may be that the neocortex has a reduced capacity to recruit the thalamus into synchronised oscillations in *Gria1*^*−/−*^ mice, or there is a disruption of intracortical connectivity^83^. It is well established that the TRN receives powerful excitatory inputs from the thalamus and the neocortex, which are, in part, mediated by AMPA receptors^76^, and which have been implicated in the generation of low- and high-frequency oscillations^75^. Notably, human studies have shown that sleep spindles often occur locally^84,85^, and their amplitude correlates with the number of EEG channels where spindles are recorded simultaneously^86,87^.

Therefore, an acknowledged caveat of our study is that local spindles may still occur in *Gria1*^*−/−*^ mice, but that they remain undetected with surface EEG recordings. Such a pattern of normal local spindle activity, in the absence of global spindles, would suggest an impairment at the thalamocortical network level, rather than a specific deficit within the TRN. Given this, it should be noted that no intracranial EEG or LFP recording has been performed in patients with schizophrenia; therefore, the possibility remains that local spindles are not in fact absent in these patients despite the lack of global EEG spindles. This might suggest the intriguing possibility that large scale network synchronisation, which is a prerequisite for reliable spindle detection at the global EEG level, is impaired in schizophrenia as a result of glutamatergic dysfunction.

### Altered REM sleep in *Gria1*^*−/−*^ mice

It has been hypothesised that REM sleep also represents an integral part of the sleep regulatory process, and is necessary to achieve efficient ‘recovery’ or network renormalisation^88–90^. Notably, REM sleep time or latency to REM sleep is affected in depression, schizophrenia and other neuropsychiatric disorders, which has led some authors to propose the total amount of REM or latency to REM sleep as endophenotypes for various psychiatric conditions^91–93^. Interestingly, we found that REM sleep episodes were longer in *Gria1*^*−/−*^ mice, which may suggest a deficiency in the dynamics of the process of network homoeostasis^88^. Notably, the tendency for longer REM sleep episodes was accompanied with a reduction of the incidence of REM sleep episodes. On the one hand, this could merely reflect the overall reduced sleep time, especially during the light period. However, it is also possible that the reduction in REM sleep episodes was compensated, at least in part, by longer REM sleep episodes.

In addition to changes in REM sleep amount and architecture, the genotypes also differed with respect to spectral EEG power in higher frequencies above 7 Hz, which was significantly lower in *Gria1*^*−/−*^ mice during REM sleep. This observation is intriguing for several reasons. First, the frequency range, which was affected as a result of GluA1 deletion in REM sleep was similar to the frequency range where the power was lower also during NREM sleep. In both cases, the effects were present in the frontal derivation only. Although it cannot be excluded that such sleep state-unspecific change in the EEG merely reflects a global dysfunction in cortical activity, we believe it is more likely that this reflects specifically the disruption in network mechanisms underlying sleep spindles. In support of this notion, the decrease of spectral EEG power was found in the frontal EEG and prior to NREM-REM transitions, where spindles are especially prominent^62^. Second, there is evidence that sleep spindles, while being manifested mostly in frequencies approximately between 10 and 15 Hz, are associated with more broad changes in spectral power, which encompasses also higher frequencies. As spindles are typically linked to the occurrence of network UP-states, it might be expected that higher frequencies would be present during intense spiking and synaptic activity associated with spindles^94^. Finally, the intriguing possibility remains that spindles, in the form of fully fledged events or subthreshold activity, occur not exclusively during NREM sleep, but are also typical for REM sleep. Consistent with this notion, it has been reported recently that sleep slow waves occur in several cortical regions during REM sleep in mice, to the extent that the two states become nearly indistinguishable^95^. Since spindles are local events, which may not be always detected reliably on the ‘global’ EEG^86,87^, the possibility remains that covert spindles do occur frequently during REM sleep, but they do not manifest as well-defined events, unless recorded in a close proximity to the source. More generally, the similar effects of GluA1 deletion on EEG power in higher frequencies, including sleep spindles, during both NREM and REM sleep, supports the notion that these two sleep states may share more similarities than it is currently appreciated^96^.

### Functional significance of impaired sleep spindles in *Gria1*^*−/−*^ mice

Our findings of reduced spindle activity in *Gria1*^*−/−*^ mice may also shed some light on the functional role of sleep spindles in normal physiology and behaviour. The purported function(s) of sleep spindles are many and varied. Sleep spindles are often discussed in the context of memory consolidation and hippocampal-to-neocortical information transfer during sleep^26–29,97^. One advantage of studying sleep and sleep EEG phenotypes in *Gria1*^*−/−*^ mice is that their behavioural phenotype has already been extensively characterised. Previous studies have shown that these mice are in fact perfectly capable of forming and retaining associative, long-term memories in numerous different memory tasks, including the water-maze and the reference memory version of the radial maze^46,51,98–100^. Importantly, under certain circumstances long-term memory is actually enhanced in these *Gria1*^*−/−*^ animals^51,101^. Thus, our finding of reduced spindle activity in these mice might argue against a straightforward role for sleep spindles in memory consolidation and long-term memory performance per se^29,102,103^, although it is important to point out that no behavioural data were collected in the current set of mice. Interestingly, however, *Gria1*^*−/−*^ mice do have an increased propensity for exhibiting habit-like behaviours (as opposed to goal-directed behaviours; e.g., refs.^104,105^), potentially consistent with the possibility that they may be less able to access or update an accurate model of their world^106^. This could potentially reflect a more nuanced role for sleep spindles in the consolidation and integration of long-term memories into a wider model of the world^103^.

### GluA1, sleep homoeostasis and attention

The higher levels of occipital SWA during baseline sleep recordings, and the persistence of more intense sleep SWA after sleep deprivation in *Gria1*^*−/−*^ mice suggests that these animals may experience a higher level of sleep pressure than wild-type controls. Previous behavioural studies in the *Gria1*^*−/−*^ mice offer an important clue for understanding their sleep phenotype. As mentioned above, *Gria1*^*−/−*^ mice are not impaired on associative, long-term memory tasks. GluA1 deletion does, however, produce a robust and reliable impairment in short-term habituation^50,51,107–109^. Thus, GluA1 plays a fundamental role in the formation of short-lasting memories that underlie a sense of familiarity, thus allowing attention to be reduced to stimuli that have been experienced recently. The adaptive value of habituation is that it reduces levels of attentional processing which are likely to have high energetic demands. It follows, therefore, that *Gria1*^*−/−*^ mice which exhibit deficits in habituation and, as a result, can display inappropriately high and prolonged levels of attention to stimuli^50,107^, will utilise greater amounts of energy and thus accumulate increased sleep pressure. This is consistent with the increase in SWA that we observe in the knockout mice in the present study. Importantly, GluA1-dependent memories that underlie habituation to recently presented stimuli are not lasting memories, and we have recently proposed that restoration of attentional performance (i.e. dishabituation) is a key behavioural endpoint of network renormalisation during sleep^106^. Thus, our study suggests that SWA and spindle activity during sleep may represent integral parts of the sleep homeostatic process, which restores the capacity for attention during subsequent wakefulness.

### Conclusions

Here we provide an important, novel link between glutamatergic dysfunction, and in particular GluA1 dysfunction, and abnormalities in sleep and sleep EEG. This may be relevant for patients with a variety of different neuropsychiatric and neurological disorders. An important question remains as to whether the deficit in spindles we report in *Gria1*^*−/−*^ mice represent a primary phenotype reflecting a direct role for GluA1 in spindle generation, or a consequence of other changes in either sleep or behaviour during preceding wake periods which then have secondary consequences for spindle activity. Nevertheless, the finding of delayed sleep onset, decreased spindles and increased REM sleep in *Gria1*^*−/−*^ mice suggests that this mouse model may have several features that make it a particularly promising tool for investigating the importance of sleep disruption and impaired synaptic plasticity/homeostasis in certain brain disorders including schizophrenia.

Finally, it has been proposed that sleep spindles may represent a promising target for selective therapeutic manipulation in a wide range of disorders, where sleep structure and/or sleep EEG are altered. Spindles represent an important sleep oscillation, tightly linked to other network activities, such as ripples and slow waves, and they are precisely regulated with respect to sleep stage, preceding sleep-wake history and time of day. Understanding the origin of spindle deficits in schizophrenia, for example, may provide important insights for understanding the sleep abnormalities in this condition. Moreover, it is possible that therapeutic approaches aimed at renormalisation of overall sleep timing and architecture will also restore sleep spindles, which could contribute to an improvement of some clinical symptoms.
